# Further Work on the Shaping of Cortical Development and Function by Synchrony and Metabolic Competition

**DOI:** 10.3389/fncom.2016.00127

**Published:** 2016-12-09

**Authors:** James J. Wright, Paul D. Bourke

**Affiliations:** ^1^Department of Psychological Medicine, School of Medicine, The University of AucklandAuckland, New Zealand; ^2^EPICentre, The University of New South WalesSydney, Australia

**Keywords:** synchronous oscillation, cortical development, synaptic development, cortical information flow, cortical computation

## Abstract

This paper furthers our attempts to resolve two major controversies—whether gamma synchrony plays a role in cognition, and whether cortical columns are functionally important. We have previously argued that the configuration of cortical cells that emerges in development is that which maximizes the magnitude of synchronous oscillation and minimizes metabolic cost. Here we analyze the separate effects in development of minimization of axonal lengths, and of early Hebbian learning and selective distribution of resources to growing synapses, by showing in simulations that these effects are partially antagonistic, but their interaction during development produces accurate anatomical and functional properties for both columnar and non-columnar cortex. The resulting embryonic anatomical order can provide a cortex-wide scaffold for postnatal learning that is dimensionally consistent with the representation of moving sensory objects, and, as learning progressively overwrites the embryonic order, further associations also occur in a dimensionally consistent framework. The role ascribed to cortical synchrony does not demand specific frequency, amplitude or phase variation of pulses to mediate “feature linking.” Instead, the concerted interactions of pulse synchrony with short-term synaptic dynamics, and synaptic resource competition can further explain cortical information processing in analogy to Hopfield networks and quantum computation.

## Introduction

Two long-standing controversies impeding the development of brain function theory concern, respectively, the functional significance of cortical synchrony, and the significance of columnar cortical organization, or its lack. It seems possible that the difficulty these problems pose singly might be overcome by finding a mutually consistent resolution, since both involve uncertainties about the way neurons code, store, and transfer information.

### Problems of synchrony

Synchrony is a favored mechanism for “binding” and cognition (Eckhorn et al., 1988, 1990; Singer, 1999, 1994) and a wide body of research infers a crucial role for synchrony in cognition (Varela et al., 2001). Although experiments usually deal with a few cells at a time, ubiquitous and widespread synchrony is evident in the EEG (Bressler et al., 1993), suggesting the assembly of currently synchronous cells can be very large.

For synchrony to mediate complicated cognitive processes, it is commonly supposed synchronous oscillation involves detailed information exchange between the linked cells, via coded pulses. Yet, simulations and analysis show that synchrony acts only to extract the in-phase component in exchanged traveling waves, rejecting out-of-phase components (Chapman et al., 2002) and can develop between linked cells activated by independent white noise inputs (Wright and Liley, 1996). The mechanism is a simple property of summing junctions, of which dendrites are an example, and this mechanism can reproduce features of synchrony seen in fundamental physiological experiments (Wright et al., 2000).

It has been recently argued that synchrony may be simply a measure of level of activation (Merker, 2016) because experiments indicate cortical gamma arising from oscillatory interaction between excitatory and inhibitory cells is self-stabilizing, and the pulse fluctuations can be accounted for entirely by this precarious excitatory/inhibitory balance, so the pulse trains could not code additional spatial and temporal information.

However, if the position and connections of each of the cells specifies their spatial and temporal inputs and outputs, rather than any property intrinsic to the cells, co-ordination of their output pulses could represent associations of spatial and temporal information *per se*, without pulse coding other than for small variations in the average levels of excitation determining the making and breaking of dynamic linkages. Dynamic changes in synaptic gain operate on appropriate time-scales for cognition as well as memory, and offer rich means for creating and breaking synchronous linkage (Wu et al., 2013).

The problem of synchrony, so formulated, becomes a problem of anatomical order, and synaptic dynamics.

### Problems of cortical organization

Uncertainties concerning the functional significance of cortical anatomical order, and its embryonic development, have persisted despite dramatic early advance (Hubel, 1981). These uncertainties compound difficulties in explanation of the role of synchrony in “binding” since they leave the properties found defined only in experimental operational terms, rather than in specified individual cell and/or network properties.

From early research in the field (Hubel and Wiesel, 1959) a key phenomenon has been the selective response of cells to specific stimuli—classically the preferred response of primary visual (V1) cortical cells to stimulus orientation (OP). OP shows organization about singularities near the center of macrocolumns (e.g., Bosking et al., 1997) with OP from 0 to 180° arranged from 0 to 360° about the singularity. In early work OP was treated as if it was a static feature, implicit in the cell's relation to its direct inputs, but more recent work shows OP and related response properties of spatial and temporal frequency preference to be interdependent (Basole et al., 2003, 2006; Issa et al., 2008).

Although the surface organization of OP in V1 in some species shows columnar order with hexagonal rotational periodicity (Muir et al., 2011; Paik and Ringach, 2011) other species, particularly small animals, exhibit little, or no sign of this ordering even in V1, although individual neurons show clearly tuned specific responses, whether, or not response maps can be clearly resolved (Girman et al., 1999). Cortical areas outside V1 show only limited, or no, apparent columnar order—so there is doubt that the pattern has any functional significance (Horton and Adams, 2005). There are suggestions of an underlying, non-random orderliness, none-the-less. Interspecies variation seems to be related to variation in size of V1 between species, with a relative constancy of the size of macrocolumns where present, independent of species (Kaschube et al., 2010; Keil et al., 2012). Systematic mapping of the response topography of mouse cortex (Garrett et al., 2014) including all higher visual areas reveals some orderliness and specialization of response, despite the absence of columns—with different areas showing variable coverage of the visual field and visual magnification. In mouse visual cortex connection probability between layer 2/3 pyramidal neurons is related to shared stimulus preference, these cells are more frequently reciprocally connected, and respond collectively to naturalistic stimuli (Ko et al., 2011), pointing to the existence of fine-scale subnetworks—although earlier in cortical development this linkage of nearby cells was not evident (Ko et al., 2013). In the squirrel, despite absence of both orderly response maps and patchiness of connections, and connections apparent between near-neighbors with different response properties, some scattered cells responded as if they were organized in pinwheels (Van Hooser et al., 2006). There is also evidence in the mouse of an orderliness of dorso-lateral geniculate projections and feedbacks from higher visual areas that is patchy, and that cell markers and differential response properties are ordered into modules, in which multiple copies are contained within the point image—the whole sharing resemblance to that of the much more orderly macaque cortex (Ji et al., 2015). All these findings suggest that there may be a common, repeating, structure to visual cortex across species, but while in some mammals the repeating structure is segregated and clearly demarcated, in others the repeating structures are intertwined.

Theories for the emergence of cortical connections at millimetric scale have focused on V1's columnar structure and its associated response maps. Following the work of Hubel and Wiesel (1959) explanations of the response maps and columnar organization assumed developmental impact of oriented visual lines (von der Malsburg, 1973; Swindale, 1982, 1992, 1996; Durbin and Mitchison, 1990; Obermayer et al., 1990; Tanaka, 1990; Miyashita and Tanaka, 1992; Grossberg and Olson, 1994) but encountered the problem that, in large species, maps of OP appear in the cortex prior to visual experience, yet also require visual experience to continue their development (Wiesel and Hubel, 1974; Blakemore and Van Sluyters, 1975; Sherk and Stryker, 1976). Recent theories, although differing amongst themselves in the cellular properties assumed, have in common an avoidance of direct-input-stimulus dependence, by applying Hebbian learning in response to long wavelength and periodic electrocortical waves, and an emphasis on long-range lateral cortical interactions rather than local direct-input tuning (Grabska-Barwinska and von der Malsberg, 2008; Bauer et al., 2014; Bednar, 2014). However, these theories still treat OP as a static property, and are aimed at explanation of order in V1, rather than providing a more general account of cortical information processing.

A related puzzle concerns the superficial patch system, composed of relatively long-range, largely excitatory (Hirsch and Gilbert, 1991; McGuire et al., 1991) patchy connections (Gilbert and Wiesel, 1979; Rockland and Lund, 1983), ubiquitous in cortex (Muir and Douglas, 2011) and, in V1, reciprocally connected and, at least in maps with limited resolution, linking areas of common OP (“like-to-like”) (Rockland and Lund, 1983; Angelucci et al., 2002). Work with a higher resolution method indicates that patch boutons are also distributed to cells of differing OP, as well as those with similar OP (Martin et al., 2014). The latter authors suggest an explanation for this disparity lies with the interrelationships of stimulus features, as found by Basole et al. (2003, 2006) and Issa et al. (2008) as opposed to the earlier views of feature responses as independent properties. Patch cells distribute their boutons most densely to other patch cells in the patch of their cell bodies of origin, distributing other boutons to further patches with regular separation, delivering fewer boutons per patch for axons with longer ranges (Binzegger et al., 2007). They do not arise or terminate near OP singularities, and instead, near singularities connections are short, diffuse, and local (Sharma et al., 1995; Yousef et al., 2001; Mariño et al., 2005; Buzás et al., 2006; Muir and Douglas, 2011). Just as for maps of response properties, there is variation of patchy connection orderliness between cortical areas, those areas higher in the hierarchy being more disordered than V1 (Muir et al., 2011).

### Our prior work and the goal of this paper

In an attempt to account for the above findings, we (Wright and Bourke, 2013) argued that in the process of embryonic development in the visual cortex, macrocolumns linked by superficial patchy connections become arranged so as to project signals to and from the surrounding cortex onto each macrocolumn in a pattern analogous to projection of a Euclidean plane onto a Möbius strip.

We were able to explain the emergence of macrocolumns and OP response maps in V1 before eye-opening, their singularities, and continuity at column margins (e.g., Bosking et al., 1997), V1 approximation to hexagonal rotational periodicity (Muir et al., 2011; Paik and Ringach, 2011), interspecies and inter-areal variation in superficial patch organization (Horton and Adams, 2005; Muir et al., 2011), relative species invariance of intersingularity distances (Kaschube et al., 2010; Keil et al., 2012), and to partly account for the emergence of ocular dominance (OD) columns (Obermayer and Blasdel, 1993). We were also able to explain the emergence of superficial patch connections “like to like” (Gilbert and Wiesel, 1989; Muir et al., 2011) with patch-sparing of macrocolumn centers (Sharma et al., 1995; Muir and Douglas, 2011), and the later absence of cortical responses to stimuli of which the subject is deprived (Blakemore and Van Sluyters, 1975).

An important property of our account is its explanation of the variation of apparent OP with stimulus orientation, angle of movement relative to orientation, object velocity, and object length found by Basole et al. (2003)—a finding contrary to expectation of fixed cell response properties. We showed that during a stimulus sweep across the visual field, the interaction of direct inputs with laterally transmitted waves generated by the stimulus at prior sites of input, could reproduce these effects via an effect similar to the Doppler shift in sound waves, and that they were equivalent to combined feature tuning of cells to stimulus orientation, spatial frequency, and temporal frequency (cp Issa et al., 2008). This means that the cell's position, its synaptic connection field, and conduction delays from lateral afferent cells determine “tuning”—not response properties intrinsic to the neuron.

Our account remained deficient in a number of ways. We did not describe the developmental sequence. We did not show that the twin cell selection pressures upon which our theory depended—ultra-small-world connectivity and maximum synchrony—were mutually compatible. We did not fully explain the periodic “skipping” of patch connections, and outside animals with columnar V1, did not explain the nature of non-columnar cortex, beyond suggesting how the columnar structure would break down beyond certain limits. However, somewhat to our surprise, in line with the findings of occasional order in non-columnar cortex described above, later findings in the somatosensory cortex (S1) (Wright et al., 2014) indicated the same sort of connection structures as postulated in V1 may be present in S1, overlapping and entangled with each other, rather than arrayed in regular columns.

We now attempt to correct these deficiencies, and propose a modular functional structure in all cortical areas, whether or not the area is columnar. In the Conclusion we argue that since this developmental account of structure depends on neither atomistic “feature” responses of cells, nor requires frequency coded signal exchanges, these properties are not necessarily essential for cognitive function at maturity. Instead, we generalize the basic assumptions of our hypotheses to fast time-scales and extensive range of metabolic competition, and show that a role for synchronous binding via dynamic synapses with potentially powerful application to cortical computation is implied.

## Review of model

### Principles and assumptions

* During early cell division cortical cells fire synchronously and organize themselves into small world configurations (Downes et al., 2012), and if prevented from firing synchronously undergo apoptosis (Heck et al., 2008). We assume the surviving cells are those forming an ensemble minimizing metabolic demand and maximizing resource uptake.
* Minimization of metabolic demand for axons implies ultra-small world (Cohen and Havlin, 2003) connectivity, requiring a power function decline of average pre-synaptic density with distance from soma. Cell types with differing axonal lengths must therefore be differentially selected to match an average presynaptic range/density power function.
* To account for survival dependence on synchrony, we assume synchrony maximizes uptake, or one or more unspecified essential resources, and that adjacent synapses cooperate in attracting resources, but compete with each other for its consumption, thus affecting synaptic dispositions. We assume synaptic adaptations, including short term depression and facilitation (Markram and Tsodyks, 1996; Zucker and Regehr, 2002), spatio-temporal facilitation (Tsukada et al., 1994), long-term potentiation (Lomo, 2003), persisting Hebbian consolidation (Hebb, 1949) and synaptic pruning (Gogtay et al., 2004), no matter how otherwise mediated, all are subject, to greater or lesser extent, to interrelated resource competitions, and are thus metabolically entangled. We note also, that local fluctuation of a limited resource would increase total synaptic Shannon entropy, and increase complexity of network pulse dynamics.
* The restricted size of dendritic trees relative to axonal trees enforces network sparsity (Liley and Wright, 1994), permitting establishment of separate, but interpenetrating, neural networks.

### Essential dynamic properties

The neural dynamics necessary for the generation of synchronous oscillation are captured in the following simplified neural field equations, as we have previously demonstrated (Robinson et al., 1998; Chapman et al., 2002; Wright, 2009, 2010). Terms in these equations are used later, but in the simulations to follow, explicit use of the dynamic equations was not necessary.

(1) $$\begin{array}{r} \phi_{p}^{\text{qr}}\left(\text{q},t\right)=g_{p}^{\text{qr}}\times Q_{p}\left(\text{r},t-x/\nu \right) \end{array}$$

(2) $$\begin{array}{r} V_{p}\left(\text{q},t\right)=G*\left(\underset{\text{r}}{\sum }\varepsilon_{p}^{\text{qr}}\phi_{p}^{\text{qr}}\right) \end{array}$$

(3) $$\begin{array}{r} Q_{p}\left(\text{q},t\right)=f_{\Sigma }\left(V_{p}\left(\text{q},t\right)\right)+E_{p}\left(\text{q},t\right) \end{array}$$

Subscript *p* = *e, i* refers to excitatory or inhibitory neurons; superscript **qr** refers to synaptic connection from **r** to **q** where **q**, **r** are cortical positions occupied by single neurons.

$$\begin{array}{r} x=|\text{q}-\text{r}| \end{array}$$

$\phi_{p}^{qr}\left(t\right)$ is the flux of pulses reaching pre-synapses at the neuron at **q**, from the neuron at **r**.

$g_{p}^{\text{qr}}$ is the synaptic density function for intra-cortical cells, composed of separate species of cells of differing characteristic axonal length.

*Q*_*p*_(**q**, *t*) is the pulse emission rate at **q**. ν is axonal conduction speed.

*V*_*p*_(**q**, *t*) are dendritic potentials generated at **q**.

$\varepsilon_{p}^{\text{qr}}\left(t\right)$ is the rate of synaptic metabolic supply, modulating each synaptic efficacy—a rate which follows the recent firing rate of a synapse. The total rate of supply is assumed to be sufficient to sustain about 50% of a given neuron's synapses at maximum efficacy.

*G* describes dendritic time-response, the convolution transforming the effective afferent synaptic flux into dendritic potentials.

*f*_Σ_(*V*_*q*_(**q**, *t*)) describes the conversion of dendritic potentials into action potentials.

*E*_*p*_(**q**, *t*) are input signals.

### Axonal metabolic competition—ultra-small world total axonal lengths

An overall power function pre-synaptic density/distance relationship for excitatory pyramidal cells can be approximated, for simplicity, by two cell populations, each with axonal trees characterized by different exponential density/distance relations.

We term these alpha and beta cells. Both types are excitatory, and it is to be understood that alpha cells are approximate to superficial patch cells, and beta cells to short-axon intracortical cells. Inhibitory cells are considered as strictly local, and are not explicitly considered other than as enabling generation of oscillation. Thus, average axonal density as a function of distance from pyramidal somas is given by

(4) $$\begin{array}{l} \rho \left({\left(x+{\lambda_{\beta }}^{-1/2}\right)}^{-2}\right)=N_{\beta }\lambda_{\beta }e^{-\lambda_{\beta }x}+N_{\alpha }\lambda_{\alpha }e^{-\lambda_{\alpha }x} \end{array}$$

where

ρ is average probability of synaptic connection between any two excitatory cells.

Subscripts α, β indicate whether the cells are of alpha or beta type.

*N*_α_ and *N*_β_ are fractions of the selected cell population composed of alpha and beta cells.

λ_α_ and λ_β_ are inverse length constants of the axonal trees of alpha and beta cells, respectively, and these vary with cortical area and species. Units of inverse length are arbitrary, so only relative axonal lengths are considered.

*g*^**qr**^ for alpha cells is represented as $\lambda_{\alpha }e^{-\lambda_{\alpha }x}$ and correspondingly for beta cells as $\lambda_{\beta }e^{-\lambda_{\beta }x}$.

$\lambda_{\beta }^{-1/2}$ displaces the power curve to the left, so that the maximum probability density of synaptic connection is equal to that of the most densely connected and closely situated cells.

### Magnitude of synchrony

It can be shown that the emergence of synchronous steady-states depends upon local excitatory/inhibitory oscillation and on the exchange of excitatory pulses at longer range (Wright, 2009, 2010). When couplings between pairs of cells are symmetrical, and average pulse rates throughout the developing cortex are uniform, synchronous equilibria emerge, with amplitude of synchronous pulse oscillations, *J*, proportional to the sum of all one-way coupling strengths.

(5a) $$\begin{array}{l} J\;\propto \;\underset{\text{q}}{\sum }\underset{\text{r}}{\sum }N_{\beta }\varepsilon^{\text{qr}}\lambda_{\beta }e^{-\lambda_{\beta }x}+N_{\alpha }\varepsilon^{\text{qr}}\lambda_{\alpha }e^{-\lambda_{\alpha }x} \end{array}$$

This sum can be decomposed into subsets of connections between pairs of alpha cells, pairs of beta cells, and pairs of alpha and beta cells.

(5b) $$\begin{array}{l} J=J_{\beta \beta }+J_{\alpha \alpha }+J_{\beta \alpha }+J_{\alpha \beta } \end{array}$$

where the subscript βα means connection from an alpha cell to a beta cell, etc.

Under the added assumption that values of ε^**qr**^ are initially random, geometrical solutions for positions of cell bodies creating maximum co-resonance, and thus maximizing each subset of *J* in Equation (5b), predict anatomically realistic patterns and response properties (Wright and Bourke, 2013) in mature columnar cortex.

## Simulation of effects on cortical development

In the simulations to follow we explore, firstly, the effects on development of ultra-small world organization as if it were the sole influence in determining the emergent cell body arrangements, next the effect of maximization of synchrony as it were the sole influence, then their combined, staged, influences, and finally, based on the simulation results for cell body positions, we consider the late optimization of antenatal synaptic distributions.

### Approximations of the power curve by exponentials

Since relative lengths of intracortical axons vary widely with species and cortical area we seek to relate this variation to differences in anatomical order. For given λ_α_ and λ_β_ the values of *N*_α_ and *N*_β_ = 1 − *N*_α_ meeting the power function pre-synaptic density/distance relation, can be found via Equation (4) by minimization of

$$\begin{array}{l} \overset{x=\infty }{\underset{x=0}{\int }}{\left[\rho \left({\left(x+{\lambda_{\beta }}^{-1/2}\right)}^{-2}\right)-\left(N_{\beta }\lambda_{\beta }e^{-\lambda_{\beta }x}+N_{\alpha }\lambda_{\alpha }e^{-\lambda_{\alpha }x}\right)\right]}^{2}dx \end{array}$$

At a distance $X=-\ln\;\left(\frac{N_{\alpha }\lambda_{\alpha }}{N_{\beta }\lambda_{\beta }}\right)/\left(\lambda_{\beta }-\lambda_{\alpha }\right)$ from their cell bodies, alpha, and beta cells have equal axonal tree density, and this distance plays a role in the simulations following.

**Figure 1:**
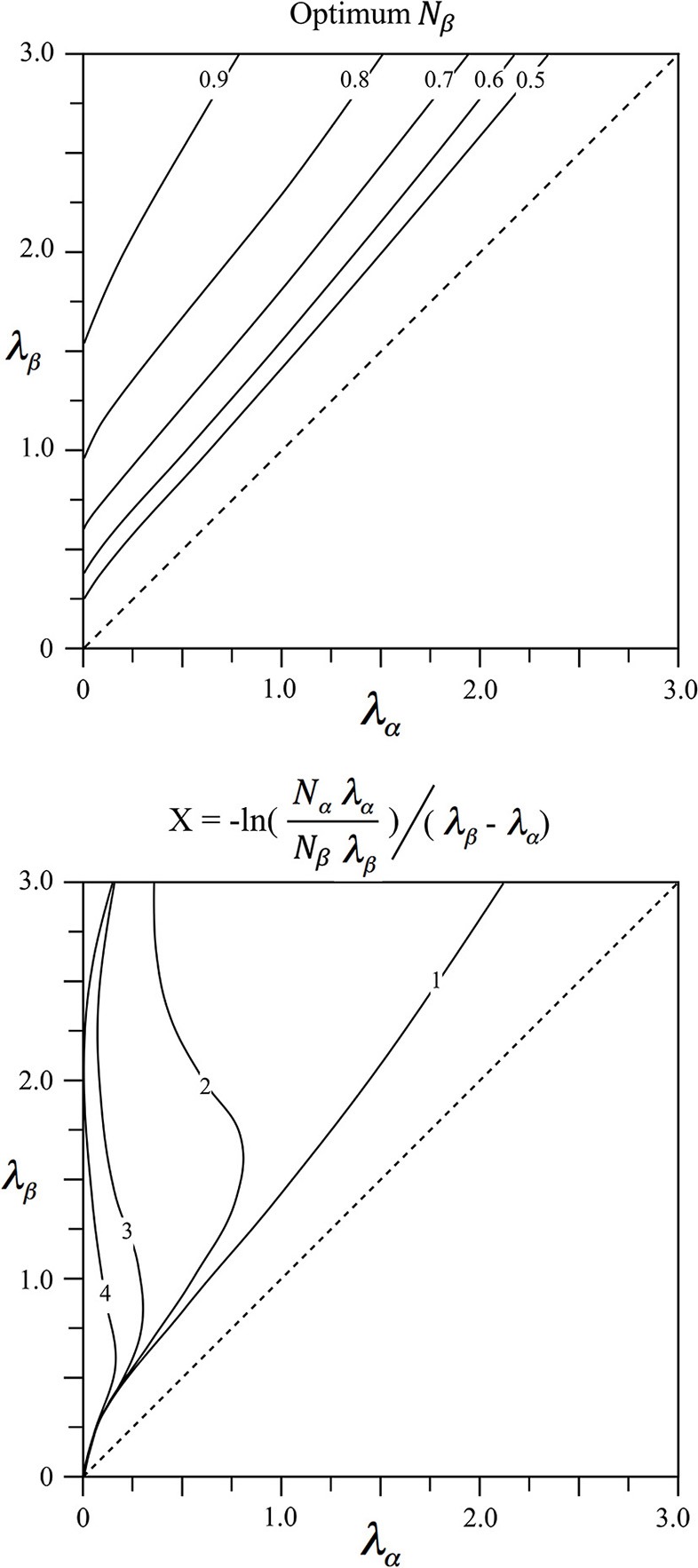
**Results of fitting two exponential axonal density functions, of inverse length constants λ_**α**_ and λ_**β**_, to a power function representing the ideal ultra-small world average density vs. distance relation**. **Top:** The fraction, *N*_β_ = 1 − *N*_α_ of cells with axons of inverse length λ_β_ required to obtain best fit. **Bottom:** The average distance, X, from cell bodies of either type at which their axonal densities are equal.

The values of *N*_β_ = 1 − *N*_α_ and *X* meeting the power relation are shown in Figure 1 as functions of λ_α_ and λ_β_. It can be seen that *N*_β_ increases to high values for alpha cells with long axons (patch cell surrogates, small values of λ_α_), and beta cells with short axons (short axon intracortical excitatory cells, high values of λ_β_). Values of *X* approach zero where λ_β_ ≈ λ_α_, on the diagonal, and tend to small value when both λ_α_ and λ_β_ are large.

### Simulation of the growth of cells maximizing post-synaptic connectivity with ultra-small world axonal length

At distances of cell separation greater than *X* the alpha cell axonal tree density exceeds that of beta cells. Therefore, to maintain the power function relation, alpha cells must preferentially make contact with other cells at distances greater than *X*, and vice-versa for beta cells.

The surplus of alpha axonal tree density over beta cell density as a function of range, and vice-versa, is given by

(6a) $$\begin{array}{r} S_{\alpha }\left(x\right)=N_{\alpha }\lambda_{\alpha }e^{-\lambda_{\alpha }x}-N_{\beta }\lambda_{\beta }e^{-\lambda_{\beta }x} \end{array}$$

(6b) $$\begin{array}{r} S_{\beta }\left(x\right)=N_{\beta }\lambda_{\beta }e^{-\lambda_{\beta }x}-N_{\alpha }\lambda_{\alpha }e^{-\lambda_{\alpha }x} \end{array}$$

Equations (6a,b) can be regarded as selection forces operating over the range of the growing axons, whereby alpha and beta cells respectively influence the probability of survival of all other cells as the network develops. By analogy, Equations (6a,b) can be used in simulation as forces pulling or pushing all other surviving cells into positions in which the forces are in equilibrium. In opposition to these forces of cell selection operating at all ranges, cell differentiation and growth can be conveniently modeled as an expansive force,

(6c) $$\begin{array}{l} G=\left(1-\sin\left(\pi \frac{x-a}{2a}\right)\right)/2\;a=0.24\;x=0\;to\;a \end{array}$$

enlarging the area occupied by each small cluster of cells.

In simulation, 8000 points were randomly distributed on a plane with toroidal bounds, and assigned randomly to alpha and beta categories with weighted likelihood in the ratio required for power function approximation at a particular pair of axonal inverse lengths. Each point was intended to represent a small group of cells. At the initiation of the simulation, the forces of selection (Equations 6a,b) were applied until the 2000th time-step, after which they were linearly scaled down until they reached zero at the 4000th time-step. The simulation was then allowed to run until the 20,000th time-step, at which time it had been verified that all cell positions were static.

In the early stages of this process it was as if, at each time-step, cells divided and either survived or died at their prior position, the surviving cells appearing at positions more consistent with an optimum distribution. As cell positions approached the optimal, and the selection forces fell to zero, the growth force (Equation 6c) enlarged the surface area covered by the points, as if cells at positions now adequate for optimum survival were now increasing in numbers.

The smoothing parameter, *a*, which is the half-value of the sine-curve maximum, had a standard setting of 0.24, as this value conveniently allowed an even packing of points in a final stable configuration.

Simulations were performed with differing initial random distribution of cells, degree of smoothing of the local repulsive force, scale of the long-range forces, and variation of the relative scale of the selection and growth forces.

**Figure 2:**
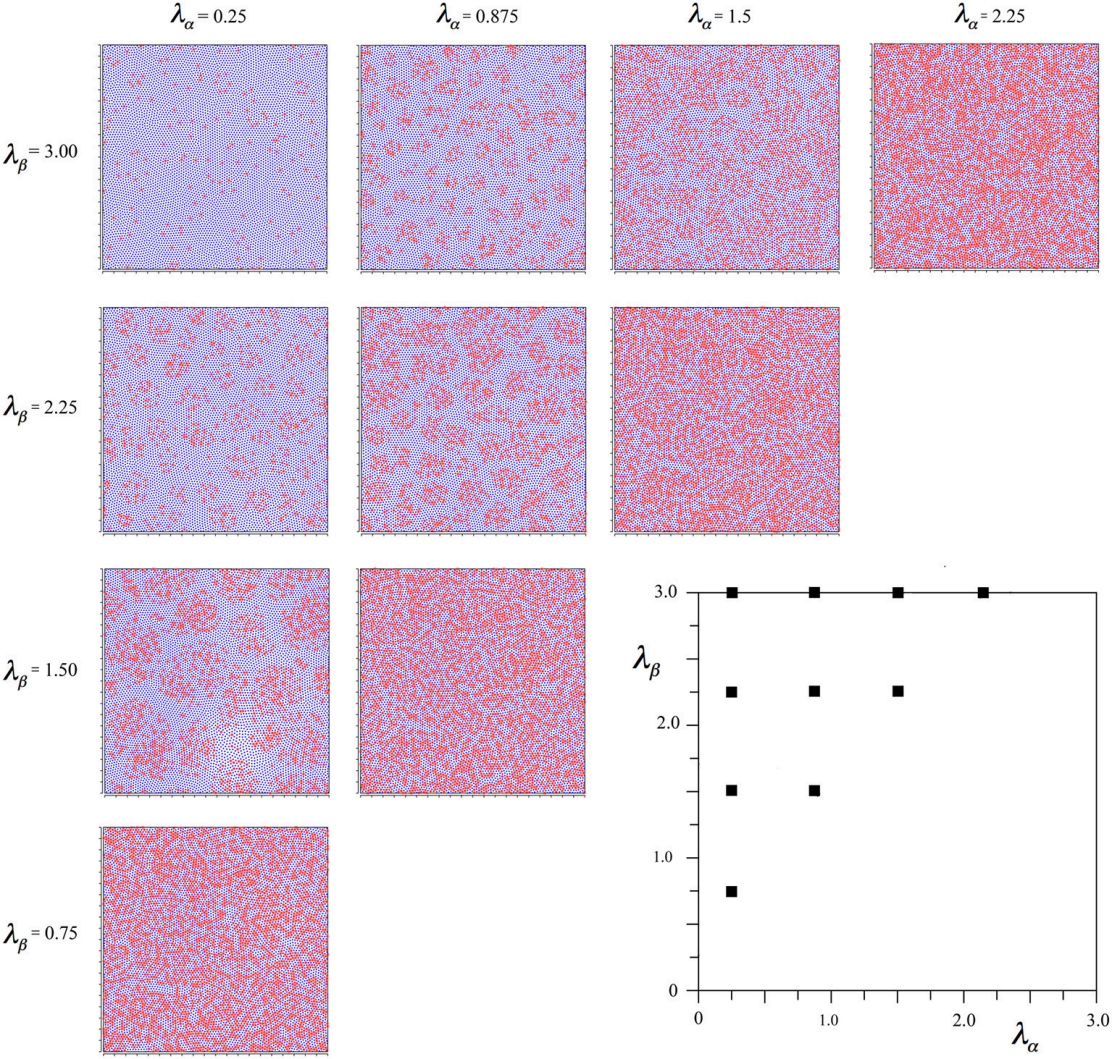
**Results of simulations showing alpha and beta cell positions maximizing ultra-small world post-synaptic connectivity, generated without regard to maximization of synchrony amplitude**. Alpha cells red, beta cells blue.

Figure 2 shows the outcomes of simulations for different combinations of λ_α_ and λ_β_, displayed in relation to the axes of Figure 1. It can be seen that where *N*_β_ and *X* are high valued, the red alpha cells are irregularly clustered in roughly rhomboidal patterns against the blue beta background, while conversely, where *N*_β_ and *X* are low valued, and alpha cell numbers approach that of beta cells, and, close to the diagonal, the clusters are blurred or non-existent.

To establish the robustness of this finding the following further comparative simulations were run. The linear scale-down of the forces of selection was varied to between 1000 and 2000, and 4000–6000. The selective forces were doubled in total gain, and halved in total gain. The short-range growth force was varied separately, by halving the force and doubling the range, and by doubling the force and halving the range. None of these variations had any significant effect on the emerging patterns except the last one, in which the simulation concluded without closure together of all the points, as expected, given the choice of parameters for Equation (6c).

### Simulation of the growth of cells with positions determined by hebbian symmetry and maximum synchrony

In contrast to the requirement to minimize axonal lengths, the maximization of synchrony in a state of equilibrum requires that signal exchange between all cells be symmetrical (whether by direct connections or by intermediate connections in the field)—and assuming a uniform average pulse rate over the field, this requires all reciprocal couplings to be equal.

**Figure 3:**
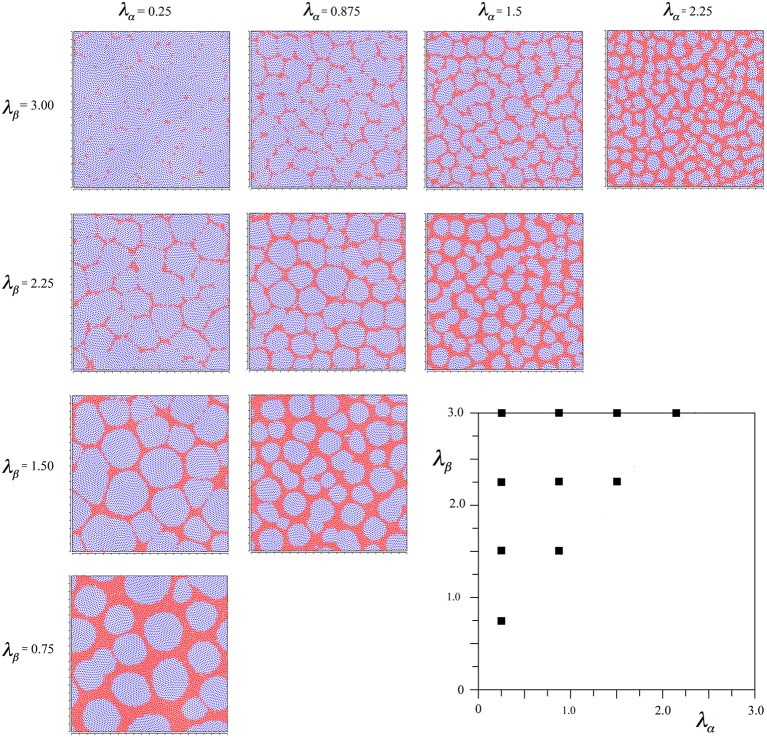
**Results of simulations showing alpha and beta cell positions maximizing synchrony amplitude generated with Hebbian connection symmetry alone**.

Connections between alpha cells and alpha cells, and beta cells and beta cells, are symmetric *a priori*, and we assume connections between beta and alpha cells attain parity because synchrony leads to Hebbian reinforcement of the weaker connections. Equations (6a,b) are then modified to

(7a) $$\begin{array}{r} S_{\alpha \alpha }\left(x\right)=N_{\alpha }\lambda_{\alpha }e^{-\lambda_{\alpha }x}-N_{\beta }\lambda_{\beta }e^{-\lambda_{\beta }x} \end{array}$$

(7b) $$\begin{array}{r} S_{\beta \alpha }\left(x\right)=N_{\alpha }\lambda_{\alpha }e^{-\lambda_{\alpha }x}-N_{\beta }\lambda_{\beta }e^{-\lambda_{\beta }x} \end{array}$$

(7c) $$\begin{array}{r} S_{\alpha \beta }\left(x\right)=N_{\alpha }\lambda_{\alpha }e^{-\lambda_{\alpha }x}-N_{\beta }\lambda_{\beta }e^{-\lambda_{\beta }x} \end{array}$$

(7d) $$\begin{array}{r} S_{\beta \beta }\left(x\right)=N_{\beta }\lambda_{\beta }e^{-\lambda_{\beta }x}-N_{\alpha }\lambda_{\alpha }e^{-\lambda_{\alpha }x} \end{array}$$

again using the subscript αβ to mean connection of a beta cell to an alpha cell, etc. With this modification, the same protocol of simulation as led to Figure 2 can be applied, and the results are shown in Figure 3.

It is seen that in all outcomes alpha cells surround beta cells in roughly hexagonal or other polygonal forms, with few breaks of their continuity. In contrast to the preceding simulation no blurring of the pattern develops near the diagonal, and the size of the sides of elements of the closed meshworks vary as the value of *X* for each λ_α_ and λ_β_ pair.

To check the robustness of these findings, the same simulation duration variations as in the preceding cases were run, and shown to produce no significant influence on the outcome. As an additional check, two further simulations were run in which the total gain of both *S*_βα_ and *S*_αβ_ were either doubled or halved—also without change in outcome.

These results reveal that the two criteria of development we applied (maximum synchrony and ultra-small-world axonal connections) are not wholly compatible.

### Results of merging initial ultra-small-world selection and later hebbian synaptic consolidation

Since the two selection criteria for development did not generate the same result, we next tried their combination, reasoning that the selections for ultra-small-world axonal lengths must precede the Hebbian stabilization of synchronous equilibrium.

**Figure 4:**
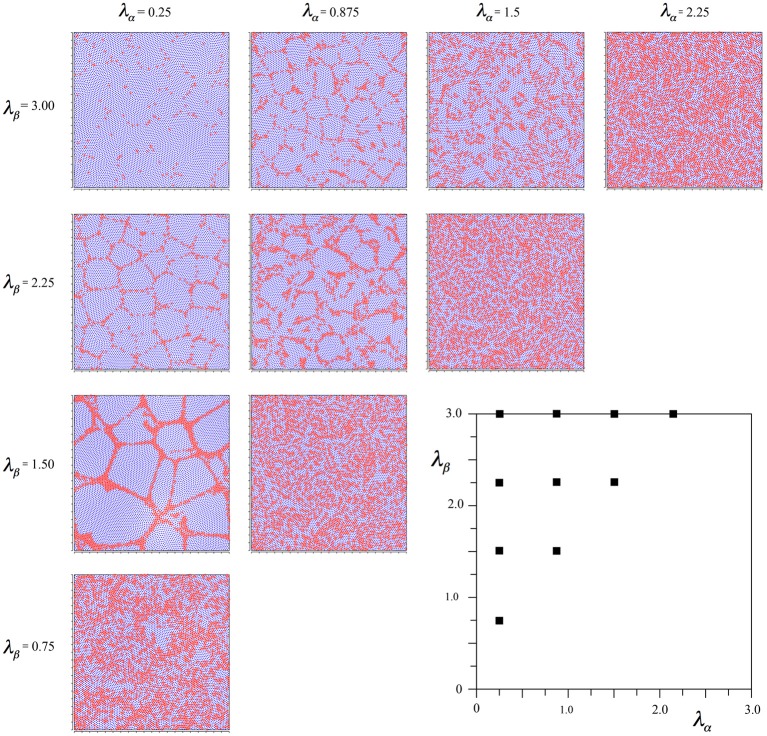
**Results of simulations showing alpha and beta cell positions maximizing ultra-small world post-synaptic connectivity early in development, then under the subsequent acquisition of Hebbian connection symmetry during the later stages of cell development**.

To achieve this effect, we started the simulations from time-step 0 using Equations (6a,b), beginning the linear scale-down of the selective forces at time-step 2000 as usual, but then changing the selection Equations to (7a–d) from 3000 until the usual conclusion. This led to the outcome seen in Figure 4.

It can be seen that the outcome is a mixture of those shown in Figures 2, 3. In common with the result in Figure 2, where *N*_β_ and *X* are low valued, and close to the diagonal, little structure is apparent at all, but where *N*_β_ and *X* are high valued, a more irregular columnar order otherwise similar to that seen in Figure 3 is present. The alpha cells are irregularly clustered in a mesh with roughly hexagonal pattern in some cases, and more irregular, larger, polygons where *X* is largest. This variation of outcome is analogous to the variation of structure in columnar and non-columnar cortex.

In comparative simulations we found, as expected, that the earlier the (Equations 7a–d) equations were initiated, the more they tended to overwhelm the earlier influence of the Equations (6a,b) equations.

### Further maximizing synchrony by optimum pre-synaptic resource distribution and development

Having shown that realistic cell body positions can arise in the simulations, we next considered consequent effects whereby distribution of synaptic resources and growth would further maximize synchrony. As previously mentioned, consistent with the power-law average presynaptic densities, the preponderance of pre-synapses made by alpha cells linking to other alpha cells must be at distance *X* or greater. Therefore, highest values of $\varepsilon_{e}^{\text{qr}}$ will become deployed, and synapses develop, at intervals *X*, or multiples of *X*, apart on their axons, thus forming reciprocal, and patchy connections on other alpha cells. This distribution is approximated by modulation of the alpha axonal tree density function with a cosine. Scaling so the function integral is unity yields the normalized synaptic density

(8a) $$\begin{array}{l} D_{\alpha \alpha }=k\cos\left(2\pi x/X\right)\lambda_{\alpha }e^{-\lambda_{\alpha }x} \end{array}$$

where $k=1+{\left(\frac{2\pi }{\lambda_{\alpha }X}\right)}^{2}$ and the positive-valued parts of the Equation (8a) then indicate favored positions of synapses.

This distribution of alpha to alpha connections will maximize the term *J*_αα_ in Equation (5b) and we will show in subsequent sections that the same periodicity of synaptic resources and synapses between alpha and beta cells leads to realistic outcomes maximizing the terms *J*_αβ_ and *J*_βα_ to Equation (5b). Beta to beta synaptic distribution follows without introduction of periodicity. Provisional synaptic density functions, including the prior Hebbian-induced symmetry, are therefore:

(8b) $$\begin{array}{r} D_{\beta \alpha }=k\cos\left(2\pi x/X\right)\lambda_{\alpha }e^{-\lambda_{\alpha }x} \end{array}$$

(8c) $$\begin{array}{r} D_{\alpha \beta }=k\cos\left(2\pi x/X\right)\lambda_{\alpha }e^{-\lambda_{\alpha }x} \end{array}$$

(8d) $$\begin{array}{r} D_{\beta \beta }=\lambda_{\beta }e^{-\lambda_{\beta }x} \end{array}$$

**Figure 5:**
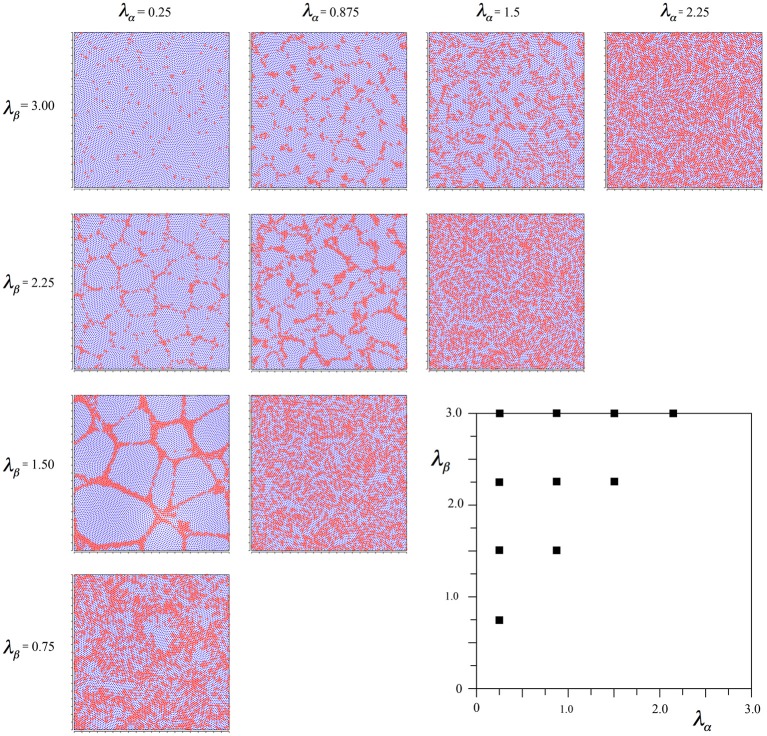
**Results of simulations showing alpha and beta cell positions maximizing ultra-small world post-synaptic connectivity under the following influence of Hebbian symmetry, and then late-stage distribution of synaptic growth resources optimizing the amplitude of synchrony**.

We introduced the positive-valued parts of Equations (8a–d) as long-range forces in a third stage in simulation, in analogy to the two prior stages resulting in Figure 4, assuming that the growth of synapses may likely also influence the late selection of surviving cells. To do this we began the simulations at 0 with Equations (6a,b), beginning the linear scale down of total long-range gains at 2000 and transferring to Equations (7a–d) at 3000 as previously, further transferring to Equations (8a–d) at 3800 before the scale down was complete. The results are shown in Figure 5.

There is little difference from Figure 4, except that alpha cells have been pulled together somewhat, into more definite clumps. These results were used in subsequent sections. Earlier initiation of equations (8a–d) resulted in more of this clumping.

We also ran a comparative simulation set using Equations (8a–d) as the only selection forces. The results did not look anatomically realistic at all. The networks of alpha cells were broken, various geometrically regular and irregular patterns appeared with no orderly change toward the diagonal. In short, the result was pathological, as might have been expected, since deployment of synaptic resources cannot begin until cell body positions are established.

## Consequent patterns of synaptic connectivity

In this section we show diagrammatically how the periodicity of resources and pre-synapses required to maximize *J*_αα_ (Equation 5b) leads concurrently, via further selection of the disposition of synapses, to maximization of *J*_αβ_ and *J*_βα_, and maximization of *J*_ββ_. That is, along with the formation of interpatch connections, goes “like to like” connections, and local connections within macrocolumns. Finally we extend the scheme to non-columnar cortex.

**Figure 6:**
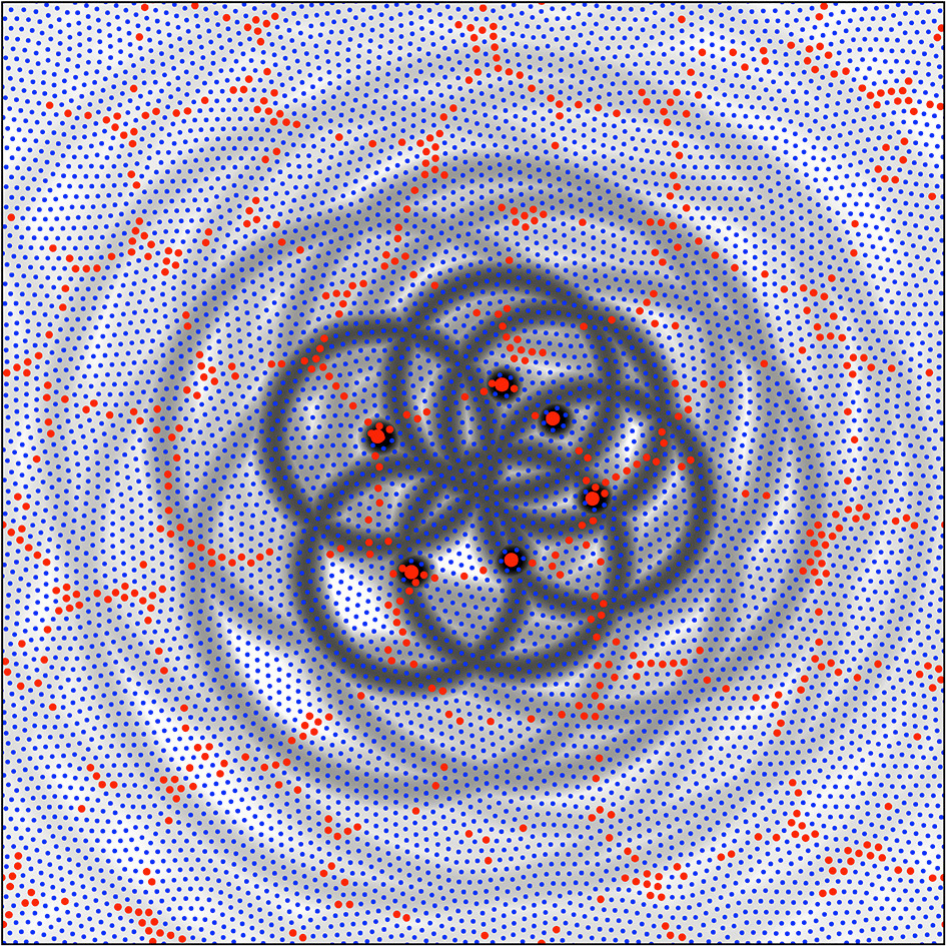
**A detail from the simulation with λ_**α**_ = 0.25, λ_**β**_ = 1.50, from Figure 5**. Alpha cells red, beta cells blue. Six alpha cells surrounding a zone of beta cells have been picked out each at distances of separation from their nearest neighbors roughly the cross-over distance, X, of relative axonal density of alpha and beta cells. The fields of potential synaptic connection of these cells with beta cells and other alpha cells (Equations 8a,b) have been emphasized in gray.

Figure 6 shows a system of six alpha cells in a portion of simulated cortex of columnar type from Figure 5. They are arranged in roughly hexagonal form about a large patch of beta cells. Rings of gray toning show the fields of periodic synaptic connection that *could* be generated by each of the alpha cells. It is important to note that not all of these connections will be made if synaptic resource is scarce, but these rings define areas in which synapses could form in proximity with the development of interpatch connections, and can do so at those positions that maximize the amplitude of synchrony, as next described.

Each alpha cell generates a field of potential connections about itself at short range, and since we can take the single cell in the simulation to represent a cluster of cells, these proximal connections are those within a patch. Each alpha cell (or patch) selected in the figure falls in the zone of synaptic connection at distance *X* from two of its six neighbors, thus generating inter-patch connections. Subsequent connections at distances 2*X*, 3*X*, etc. fall near other alpha cells permitting “skipping” patch connections.

**Figure 7:**
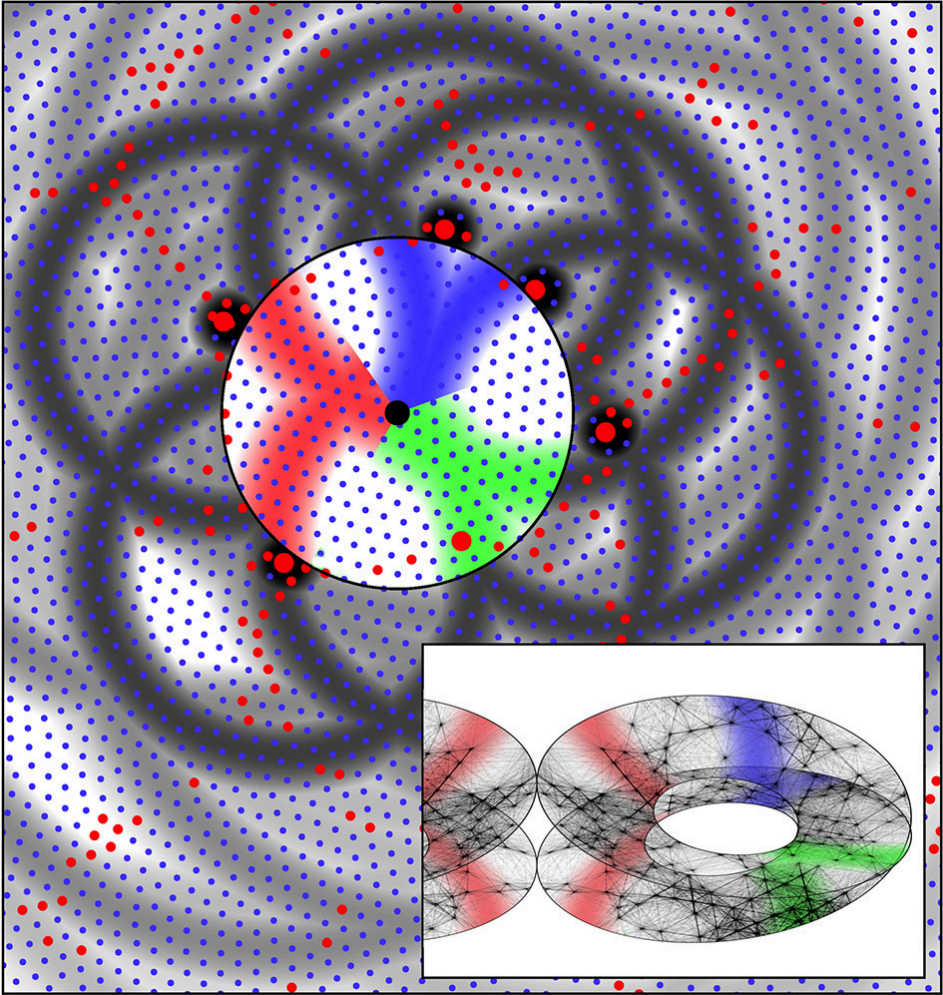
**The same system of six alpha cells and their fields of potential synaptic connection as in Figure 6**. Connections to beta cells within the central zone are now colored red, green, and blue according to their origins from diametrically opposite alpha cells. Consequent to restriction of synaptic resources, connections have become established over only half the potential field, and these are arranged so diametrically opposite alpha cells make connections outwards at similar angles from a “singularity.” The inset shows the corresponding form of connections among the beta cells within the zone. Concurrently, the beta cells have established connections so they form a connected system analogous to a Möbius strip. This synergic distribution of resources to alpha-beta and beta-beta connections is that which maximizes synchronous resonance of the system. The inset also shows the mirror-image arrangement of connections in an adjacent column of beta cells.

It can also be seen that the rings of potential synaptic contact at distance *X* from the alpha cell bodies cut through the central cluster of beta cells, passing close to the center. Rings of potential synaptic positions generated from the diametrically opposite alpha cells pass close to each other. Selection from among these potential sites of alpha to beta synapses and reciprocal beta to alpha connections can take place, and can produce an organization similar in appearance to a cortical macrocolumn with an OP singularity at its center, as follows in Figure 7.

Figure 7 shows the same system of cells as in Figure 6, marked to show, in color, emergent connections within the group of beta cells that will maximize, by co-resonance, the amplitude of synchrony. A half of the zones of potential synaptic connection to beta cells from pairs of alpha cells roughly diametrically opposite each other, have been highlighted in the same color, and connections from opposite sides are made radially in the same direction from the beta cell group's center.

The inset shows that if the beta cells themselves form into a re-entrant loop of connected cells, analogous to a three dimensional representation of a Möbius strip (for reasons next given) then the formation of alpha to beta synapses and the reciprocal beta to alpha synapses, in the red, blue, and green colored domains, form a connection system maximizing the synchronous resonance between the beta cells and the surrounding alpha network—i.e., maximizing *J*_αβ_ and *J*_βα_ —since they form a map of nearest-connected-neighbor alphas onto nearest-connected-neighbor betas. This map of connections can be represented as

(9) $$\begin{array}{l} P\left(x,y\right)↔p\left(x,y,z\right) \end{array}$$

where *P*(*x, y*) are the locations of patch cells at horizontal co-ordinates on the cortical surface, mapped by synapses to cells within the macrocolumn at positions *p*(*x, y, z*) and

$$\begin{array}{l} p_{x}=|p|\cos\left(\pm \vartheta +\phi \right)\\ p_{y}=|p|\sin\left(\pm \vartheta +\phi \right)\\ p_{z}=|p|\sin\left(\left(\pm \vartheta +\phi \right)/2\right) \end{array}$$

*z* is the cortical depth, ϑ is polar angle, ± indicates the chirality of the map, and ϕ its orientation.

Arrangement of the beta-to-beta connections in the Möbius-strip-like manner is that which will maximize *J*_ββ_, since, all connections being sparse, unconnected cells can be closely situated to each other in space, while established connections are between cells packed as closely as possible—yet this mapping is one between cell connection systems with topological identity, as required for maximum co-resonance. Thus, the beta cells can form a tight network, maximizing *J*_ββ_ and concurrently permitting the maximization of *J*_αβ_ and *J*_βα_.

**Figure 8:**
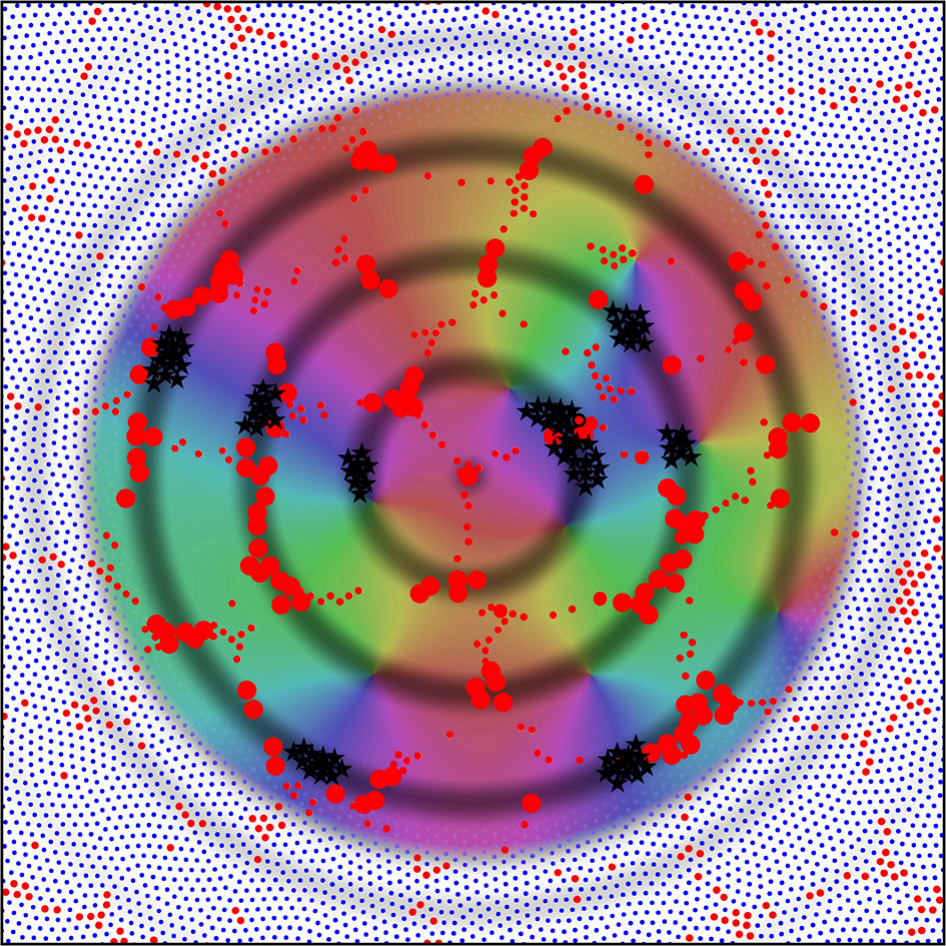
**Utilizing the analogy of the connections shown in Figure 7 to the distribution of OP from 0 to 180° over 360° about an OP singularity, an emergent pattern of OP is shown by arrangement in adjacent systems of alpha cells and their enclosed beta cells**. This pattern in centered on one of the original six alpha cells in Figure 6. Those alpha cells to which the central cell makes strong connection are shown as enlarged red filled circles, and those beta cells with common OP connected to the central alpha are marked with black stars. These patterns are those that maximize synchronous resonance, and resemble patch cell connections, and “like to like” connections, respectively.

Figure 8 shows consequent alignment of maps in adjacent macrocolumns, the structure of extended patch cell connections, and the formation of “like to like” connections. The image is centered on a single alpha cell from the original set of six, with the darkened rings of potential connectivity surrounding it. Color wheels have been superimposed, each fitting within a macrocolumn, and vector smoothed to continuity with each other. These are arranged so that each forms an approximate mirror image of its neighbor. Each represents a connection system like that shown for a single macrocolumn in Figure 7. The rationale for the arrangement is that as multiple maps form in adjacent macrocolumns, they must be arranged with reverse chirality so that homologous points in adjacent maps are closely situated, and, consequently, beta cell co-resonance is further maximized. Reversal of chirality in adjacent maps requires only that beta-alpha synaptic connections—like those colored red, green, and blue in Figure 7—be deployed in opposite direction outwards from the centers of the macrocolumn to those shown. The overall resemblance to a cortical map of OP is apparent. The analog of OP singularities can be seen at approximately 1 o'clock, 4 o'clock, and 9 o'clock from the central alpha cell.

The rings of potential connection outward from the alpha cell intersect some other alpha cells in a regular, periodic, manner. The development of synapses on these intersected alpha cells, but not on all nearby alpha cells, accounts for the spatially periodic nature of patch cell connections, while where the rings of potential connection pass through beta cells in areas of similar “OP” in different maps, preferential synapse formation forms “like to like” connections, shown here as black stars. Although not indicated in this figure, the repetition of mirror reversal in adjacent maps in one direction can account for the prolongation of patch connections mainly in one direction, as is seen in experimental data (Bosking et al., 1997).

It may be mentioned in passing that, as the simulations show, the most efficient packing of cells is achieved by hexagonal arrays of alpha cells surrounding beta cells. An exception would occur when the ratio of beta cells to alpha cells is approximately π/4. Then a uniform square network of alpha cells would be possible and the adjacent OP maps would be able to be arranged exactly in mirror reflection, producing a stable map with the properties of OD columns.

**Figure 9:**
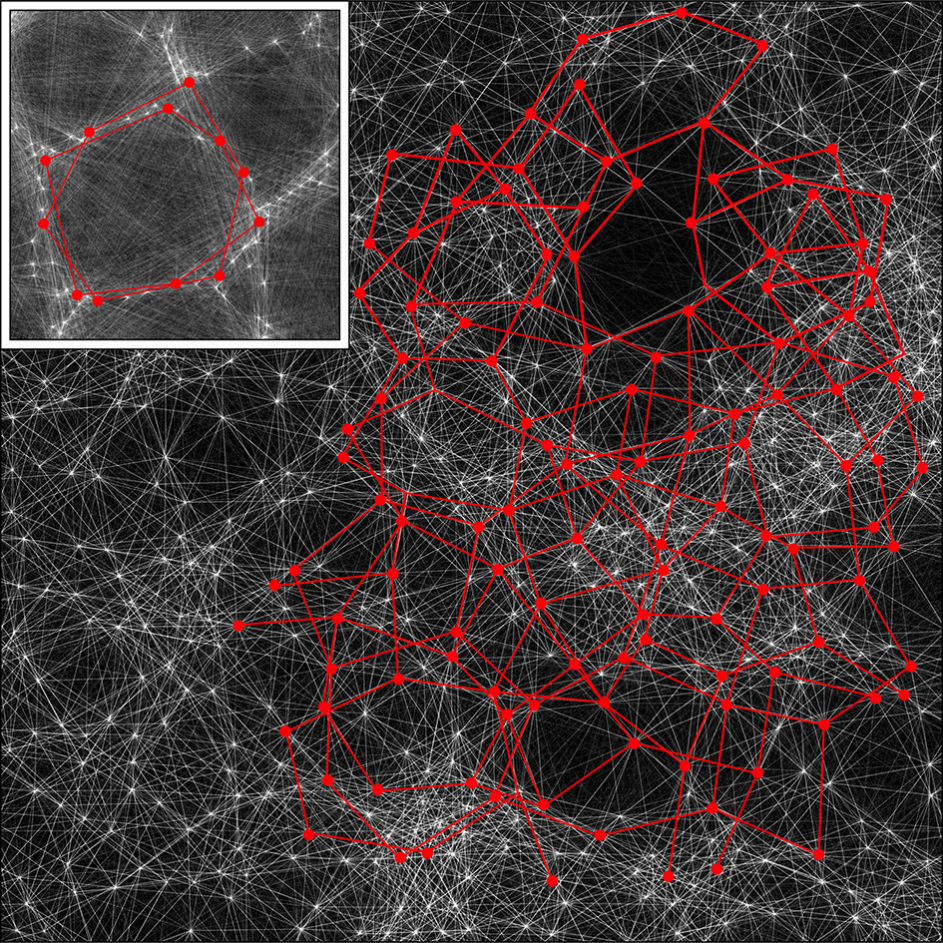
**A section of the simulation result obtained with λ_**α**_ = 2.25, λ_**β**_ = 3.00 from Figure 4, magnified by a factor of 2.5, with the detail used in Figure 5 inserted as an inset**. By rescaling, the cross-over distance, X, is normalized between the section and the inset. Alpha cell connections approximately distance X from near neighbors are then picked out by highlighting in red, in both the section and the inset, showing that similar connection structures can exist in both, but with marked interweaving in the “non-columnar” case.

The schema for columnar cortex can be readily extended to non-columnar cortex, since sparse synaptic networks architectures can be interwoven with each other. Figure 9 shows this.

A small portion of a simulation in Figure 5 that does not exhibit obvious organization into columns is shown, with all the alpha cells as white dots and their interconnections as white lines. Inset is the same portion of simulation, showing columnar structure, used in the prior figures, with the alpha cells and their connections similarly rendered to the main figure. The main figure has been scaled so that distance *X* is normalized to be equal in the main figure and the inset.

In both the main figure and the inset, alpha cells and their connections have been highlighted in red to show systems of closed connections with distances between nearest neighbors equal to *X*. It can be seen that a myriad of such networks, each intertwined with other networks, are possible in the non-columnar case, and in the inset case the same is true, but in the latter the intertwined networks are necessarily close to superposition.

Thus, as a consequence of the sparsity of neural connections, similar networks can exist in either columnar or non-columnar cortex, the only difference being the differing balance between optimizing ultra-small-world axonal connections vs. Hebbian-symmetric equilibrium. Because of the intertwining, OP would appear to be “pepper and salt” in the non-columnar case, and relatively orderly in the columnar case. At some places in the non-columnar cortex (e.g., near the top right of the image) there are occasional areas approaching the orderliness of columnar cortex.

## Conclusion

These simulation outcomes support our prior hypotheses on organization of columnar cortex. They explain the development of cortex in sequential, partially antagonistic but ultimately synergistic steps, and extend the scope to non-columnar cortex. The differences between columnar and non-columnar cortex are shown to be attributable to variable compromise between ultra-small-world axonal length optimization, and maximization of synchrony, depending upon the relative lengths of patch axons and short-axon cells in the cortical area. It appears the entire cortex may be a matrix of overlapping and intertwined elements, in which each element is a folded central topographic map with patch projections to and from the surrounding cortex—an arrangement within which metabolic efficiency and speed of communication reach optimum.

The results also explain how non-columnar cortex can exhibit small areas with properties like columnar cortex, against a predominantly apparently random background pattern (Van Hooser et al., 2006; Ko et al., 2011, 2013; Garrett et al., 2014; Ji et al., 2015), and, in the same way, provides an improved explanation for our earlier findings in somatosensory cortex (Wright et al., 2014). We have also now accounted, via Equation (8), for the periodic form of patch connections and inter-patch intervals (Binzegger et al., 2007).

With the notable exception of the Möbius configuration of synapses within each element's short-axon cells, all the synaptic connections in the theoretical account have already been demonstrated by direct anatomical means. The most rigorous anatomical test of the model therefore depends on whether or not the closure of synaptic connections into the Möbius configuration, shown in Figure 7 (inset), can also be directly demonstrated, rather than indirectly inferred. That this has not already been directly demonstrated is not surprising, since very many synaptic connections would need to be charted in a single experiment. Techniques for the tracing and analysis of very numerous synaptic contacts are currently being developed (e.g., Kasthuri et al., 2015) and, perhaps linked to modifications of algorithms to extract paths of connection (Reimann et al., 2015), may prove applicable.

A simpler test is to relate the ratio of lengths of patch cell axons and cells with short axons to the degree of columnar organization in different cortical areas and species. To the best of the authors' knowledge there are not readily available and sufficiently exhaustive data, cortex and species-wide, to make this test. A complication arises since we have simplified the many classes of excitatory cortical neurons into two classes. Since, realistically, a larger variety of cell types must be considered, the ratio of lengths might be converted to a measure of skewness of axonal length distribution, to enable the test.

Further tests of hypothesis are possible along the lines of our earlier reported experiment (Wright et al., 2014) with single cell receptor field recordings made from near-vertical penetrations, and by more detailed analysis of response to visual moving objects (cp. Basole et al., 2006) throughout cells surrounding a singularity, as was diagramed in our earlier theoretical paper (Wright and Bourke, 2013).

If this model is found to be consistent with further anatomical findings, then it will become imperative to undertake extensive modeling of likely processes underlying the assumed metabolic uptake effect of synchrony, and of the biochemistry of the assumed metabolic competitions. We have avoided speculation on these issues for present purposes, although we are aware of the large and incomplete body of relevant data available. However, regardless of the biochemical detail, the time-constants and range of the metabolic exchanges are of importance to the range of application of the theory. If the time-constants are slow, and the range of metabolic exchanges short, then the theory's application would be confined to slower, developmental, processes. However, if these processes are fast, and occur over long ranges, then there are important implications for the role of synchrony in information processing generally, as argued further below. We consider first the implications for later cortical organization on the time-scale of long-term learning.

The late antenatal configuration of each of our hypothetical elements provides a ground state, with maximum potential information storage capacity, which is both an initial organizational framework for learning, and a default state for forgetting. As postnatal learning begins, the antenatal organization would be progressively overwritten. The organization of the connection overwriting can be inferred from our explanation of object representation and feature tuning in V1, as previously cited in the Introduction (Wright and Bourke, 2013). As a property of the antenatal evolution of the connections, any small segment of a moving visual object, projected to the visual cortex as a stimulus pattern, *O*_*P*_, is transmitted with axonal delays to each cortical column surrounding an OP singularity as a mapped transformed pattern, *O*_*p*_

(10) $$\begin{array}{l} O_{P}\left(K_{x},K_{y},{\text{v}}_{x}\right)\left(t\right)\to O_{p}\left(k_{x},k_{y},\omega \right)\left(t+|P-p|/\nu \right) \end{array}$$

where **v**_*x*_ is the velocity of the stimulus pattern moving over the visual cortex along axis *x* to cross the classical receptive field of the macrocolumn, *K*_*x*_, *K*_*y*_, *k*_*x*_*k*_*y*_ are dominant spatial frequencies of the visual object's projection and mapped response respectively, ω is the temporal frequency of response, ν is the speed of electrocortical wave transmission, and

$$\begin{array}{lll} k_{x} & \propto & \frac{\nu }{\nu \pm {\text{v}}_{x}}K_{x}\\ k_{y} & \propto & K_{y}\\ \omega =K_{x}{\text{v}}_{x} \end{array}$$

Thus, the four dimensional world of moving objects becomes represented as stored learning within the four dimensional space defined by the complex vector pairs, *P* and *p*. All neurons stimulated by a single moving object can then enter into synchronous oscillation, and thus establish Hebbian interconnections. The antenatal organization thus provides a reference framework—a dimension-reduction to the flattened cortical surface—for postnatal information storage, upon which a single stimulus is represented in multiple local transforms within the primary cortex. Consequently representations of different objects each with some shared spatio-temporal features can become linked within overlapping separate synchronous sets, as multiple partial correlations, permitting overall consistency of associations so formed. The antenatal reference framework differs from an initial system of random connections, because the initial connectivity is a map of visual (or other sensory) space in which covariance is everywhere declining with increasing distance in space and time. By similar processes operating over interareal forward and backward, divergent and convergent, projections, superpositions of signals from the primary cortices would be created, and could achieve increasingly abstract associative synaptic representations in association cortex, mediating between the primary sensory and all efferent connections of cortex. All output (motor) cortices also require the same four dimensional reference system, to generate motion in motor space, and since association cortex must ultimately transform inputs to outputs, provision of a similar four dimensional reference frame throughout the cortex offers a universal, dimensionally commensurate, mode of information representation and manipulation. Thus, the ground state, overwritten locally by cross-links, and between areas as superpositions with translations, rotations and reversals of chirality, offers rich means for the construction of associations.

There are wider implications still, if metabolic competition between synapses is fast, on the time-scale of fast synaptic dynamics, and operates by rapid diffusion over ranges large compared to a synapse. As mentioned in the Introduction, there is no need for the exchange of frequency-coded information in pulses, outside the time frame set by dynamic synapses. The assumption was made in Equation (5a) that average pulse activity is uniform and synaptic connectivity symmetrical. However, synchronous equilibria are permitted more generally, when for all pairs of cells $\varepsilon^{qr}\times \;{\bar{\varphi }}^{qr}=\;\varepsilon^{rq}\times \;{\bar{\varphi }}^{rq}$, (see Equation 2), so a multitude of synchronous states are made possible by compensated adjustment of pulse rates and synaptic efficacies. Shifting patterns of subcortical activation, and adiabatic variations of inputs between cortical areas, would continuously modulate access to domains in the state-space of possible equilibria—and we can write a corresponding synaptic energy function

(11) $$E(\bar{\varphi })=\sum_{i,j=1}^{i,j=N}(\varepsilon_{i}{\bar{\varphi }}_{i}+B_{ij}{\bar{\varphi }}_{i}{\bar{\varphi }}_{j})\;j\neq i$$

where *i, j* are synaptic connections between some **q** and **r**, *N* is the total number of synapses, and *B*_*ij*_ measures the degree to which the *i*−*th* and *j*−*th* synapses share joint success in the acquisition of the critical metabolites. Where *E*(φ) is at a maximum {ε_*i*_, *B*_*ij*_} are in steady-states of synaptic adaptation and pulse exchange. That is, the set of all possible synchronous states is the set of ways synaptic resources and cell pulses can be aligned to steady state in concert. Equation (11) has analogous form to the energy function

(12) $$\begin{array}{l} E\left(S\right)=\overset{i,j=N}{\underset{i,j=1}{\sum }}\left(h_{i}S_{i}+J_{ij}S_{i}S_{j}\right)\;j\neq i \end{array}$$

applicable to learning in Hopfield networks (Amit, 1989), where *S* are inputs, *h* and *J* are determined by a learning rule, and minima of *E*(*S*) define point attractor basins. Thus, in the cortex, stable patterns of synchronous oscillation are equivalent to convergence to point attractors in attractor neural networks. If synaptic resources are rapidly readjusted and entangled at considerable range, then very rapid and powerful parallel computations become possible, since the same equation applies to the execution of quantum computations, where *E*(*S*) is minimized to achieve solution, *S* are quantum probabilities, and *h* and *J* are weights applied to probabilities and joint probabilities, and constitute the program (Dwave, 2013)—a program that concludes when uncertainty in the quantum states is removed. There is thus some analogy between quantum uncertainty and noisy initial synaptic states, and quantum entanglement with conserved sum of probabilities vs. metabolic entanglement of synapses with limited, constant, total resource. Both are capable of executing a program by converging to a stable state. If the metabolic entanglement range and speed are great, then rapid convergence to global attractors rather than distraction by local minima (as Hopfield nets are prone to) may take place, in analogy to quantum tunneling.

Since capacity for computation does not imply the learning of behaviorally useful computations, we must further suppose supervision of the cortex by limbic system interventions on activation and neuromodulation, as initially proposed in the “Triune Brain” concept (Maclean, 1973), so that transient values of {*B*_*ij*_} are consolidated if they facilitate goal-directed limbic activity.

Synchronous states could thus mediate the storage of information and its release when suitably activated, and lead to evolution of individual, novel—essentially “creative”—behavioral adaptations.

## Author contributions

JW and PB are jointly responsible for this work.

### Conflict of interest statement

The authors declare that the research was conducted in the absence of any commercial or financial relationships that could be construed as a potential conflict of interest.

## References

[B1] Amit D. J. (1989). Modelling Brain Function. The World of Attractor Neural Networks. Cambridge: Cambridge University Press.

[B2] Angelucci A. Levitt J. B. Lund J. S. (2002). Anatomical origins of the classic receptive field and modulatory surround field of single neurons in macaque visual cortical area V1. Prog. Brain Res. 136, 373–388. 10.1016/S0079-6123(02)36031-X 12143395

[B3] Basole A. Kreft-Kerekes V. White L. E. Fitzpatrick D. (2006). Cortical cartography revisited: a frequency perspective on the functional architecture of visual cortex. Prog. Brain Res. 154, 121–134. 10.1016/S0079-6123(06)54006-3 17010706

[B4] Basole A. White L. E. Fitzpatrick D. (2003). Mapping of multiple features in the population response of visual cortex. Nature 423, 986–990. 10.1038/nature01721 12827202

[B5] Bauer R. Zubler F. Pfister S. Hauri A. Pfeiffer M. Muir D. R.. (2014). Developmental self-construction and configuration of functional neocortical networks. PLoS Comput. Biol. 10:e1003994. 10.1371/journal.pcbi.1003994 25474693 PMC4256067

[B6] Bednar J. A. (2014). Hebbian learning of the statistical and geometrical structure of visual input, in Neuromathematics of Vision. Lecture Notes in Morphogenesis, eds Citti G. Sarti A. (Berlin; Heidelberg: Springer-Verlag), 335–366.

[B7] Binzegger T. Douglas R. J. Martin K. A. C. (2007). Stereotypical bouton clustering of individual neurons in cat primary visual cortex. J. Neurosci. 27, 12242–12254. 10.1523/JNEUROSCI.3753-07.2007 17989290 PMC6673271

[B8] Blakemore C. Van Sluyters R. C. (1975). Innate and environmental factors in the development of the kitten's visual cortex. J. Physiol. 248, 663–716. 10.1113/jphysiol.1975.sp010995 1151843 PMC1309546

[B9] Bosking W. H. Zhang Y. Schofield B. Fitzpatrick D. (1997). Orientation selectivity and the arrangement of horizontal connections in tree shrew striate cortex. J. Neurosci. 17, 2112–2127. 9045738 10.1523/JNEUROSCI.17-06-02112.1997 PMC6793759

[B10] Bressler S. L. Coppola R. Nakamura R. (1993). Episodic multiregional cortical coherence at multiple frequencies during visual task performance. Nature 366, 153–156. 10.1038/366153a0 8232553

[B11] Buzás P. Kovács K. Ferecskó A. S. Budd J. M. L. Eysel U. T. Kisvárday Z. F. (2006). Model-based analysis of excitatory lateral connections in the visual cortex. J. Comp. Neurol. 499, 861–881. 10.1002/cne.21134 17072837

[B12] Chapman C. L. Bourke P. D. Wright J. J. (2002). Spatial eigenmodes and synchronous oscillation: coincidence detection in simulated cerebral cortex. J. Math. Biol. 45, 57–78. 10.1007/s002850200141 12140691

[B13] Cohen R. Havlin S. (2003). Scale- free networks are ultra-small. Phys. Rev. Lett. 90:058701. 10.1103/PhysRevLett.90.058701 12633404

[B14] Downes J. H. Hammond M. W. Xydas D. Spencer M. C. Becerra V. M. Warwick K.. (2012). Emergence of a small-world functional network in cultured neurons. PLoS Comput. Biol. 8:e1002522. 10.1371/journal.pcbi.1002522 22615555 PMC3355061

[B15] Durbin R. Mitchison G. (1990). A dimension reduction framework for understanding cortical maps. Nature 343, 644–647. 10.1038/343644a0 2304536

[B16] Dwave (2013). An Elementary Introduction to Quantum Computing. Available online at: www.dwavesys.com

[B17] Eckhorn R. Bauer R. Jordon W. Brosch M. Kruse W. Monk M.. (1988). Coherent oscillations: a mechanism of feature linking in the in the visual cortex? Biol. Cybern. 60, 121–130. 10.1007/BF00202899 3228555

[B18] Eckhorn R. Reitboeck H. J. Arndt M. Dicke P. (1990). Feature linking via synchronization among distributed assemblies: simulations of results from cat visual cortex. Neural Comput. 2, 293–307. 10.1162/neco.1990.2.3.293

[B19] Garrett M. E. Nauhaus I. Marshel J. H. Callaway E. M. (2014). Topography and areal organization of mouse visual cortex. J. Neurosci. 34, 12587–12600. 10.1523/JNEUROSCI.1124-14.2014 25209296 PMC4160785

[B20] Gilbert C. D. Wiesel T. N. (1979). Morphology and intracortical projections of functionally character- istic neurons in cat visual cortex. Nature 280, 120–125. 10.1038/280120a0 552600

[B21] Gilbert C. D. Wiesel T. N. (1989). Columnar specificity of intrinsic horizontal and corticocortical connections in cat visual cortex. J. Neurosci. 9, 2432–2442. 2746337 10.1523/JNEUROSCI.09-07-02432.1989 PMC6569760

[B22] Girman S. V. Sauvé Y. Lund R. D. (1999). Receptive field properties of single neurons in rat primary visual cortex. J. Neurophysiol. 82, 301–311. 10400959 10.1152/jn.1999.82.1.301

[B23] Gogtay N. Giedd J. N. Lusk L. Hayashi K. M. Greenstein D. Vaituzis A. C.. (2004). Dynamic mapping of human cortical development during childhood through early adulthood. Proc. Natl. Acad. Sci. U.S.A. 101, 8174–8179. 10.1073/pnas.0402680101 15148381 PMC419576

[B24] Grabska-Barwinska A. von der Malsberg C. (2008). Establishment of a scaffold for orientation maps in primary visual cortex of higher mammals. J. Neurosci. 28, 249–257. 10.1523/JNEUROSCI.5514-06.2008 18171942 PMC6671147

[B25] Grossberg S. Olson S. J. (1994). Rules for the cortical map of ocular dominance and orientation columns. Neural Netw. 7, 883–894. 10.1016/S0893-6080(05)80150-9

[B26] Hebb D. O. (1949). The Organization of Behavior. New York, NY: John Wiley.

[B27] Heck N. Golbs A. Riedemann T. Sun J.-J. Lessmann V. Luhmann H. J. (2008). Activity dependent regulation of neuronal apoptosis in neonatal mouse cerebral cortex. Cereb. Cortex 18, 1335–1349. 10.1093/cercor/bhm165 17965127

[B28] Hirsch J. A. Gilbert C. D. (1991). Synaptic physiology of horizontal connections in the cat's visual cortex. J. Neurosci. 11, 1800–1809. 1675266 10.1523/JNEUROSCI.11-06-01800.1991 PMC6575415

[B29] Horton J. C. Adams D. L. (2005). The cortical column: a structure without a function. Philos. Trans. R. Soc. Lond. B Biol. Sci. 360, 837–862. 10.1098/rstb.2005.1623 15937015 PMC1569491

[B30] Hubel D. H. (1981). Evolution of Ideas on the Primary Visual Cortex, 1955-1978: A Biased Historical Account. Open Access at the Nobel Institute, 8 December 1981.

[B31] Hubel D. H. Wiesel T. N. (1959). Receptive fields of single neurones in the cat's striate cortex. J. Physiol. 148, 574–591. 10.1113/jphysiol.1959.sp006308 14403679 PMC1363130

[B32] Issa N. P. Rosenberg A. Husson T. R. (2008). Models and measurements of functional maps in V1. J. Neurophysiol. 99, 2745–2754. 10.1152/jn.90211.2008 18400962

[B33] Ji W. Gamanut R. Bista P. Burkhalter A. (2015). Modularity in the organization of primary visual cortex. Neuron 87, 632–634. 10.1016/j.neuron.2015.07.004 26247867 PMC4529541

[B34] Kaschube M. Schnabel M. Löwel S. Coppola D. M. White L. E. Wolf F. (2010). Universality in the evolution of orientation columns in the visual cortex. Science 330, 1113. 10.1126/science.1194869 21051599 PMC3138194

[B35] Kasthuri N. Hayworth K. J. Berger D. R. Priebe C. E. Pfister H. Lichtman J. W. (2015). Saturated reconstruction of a volume of neocortex Cell 162, 648–661. 10.1016/j.cell.2015.06.054 26232230

[B36] Keil W. Kaschube M. Schnabel M. Kisvarday Z. F. Löwel S. Coppola D. M. (2012). Response to comment on “Universality in the evolution of orientation columns in the visual cortex”. Science 336, 413 10.1126/science.1206416 PMC3138194 21051599

[B37] Ko H. Cossell L. Baragli C. Antolik J. Clopath C. Hofer S. B.. (2013). The emergence of functional microcircuits in visual cortex. Nature 496, 96–100. 10.1038/nature12015 23552948 PMC4843961

[B38] Ko H. Hofer S. B. Pichler B. Buchanan K. A. Sjöström P. J. Mrsic-Flogel T. D. (2011). Functional specificity of local synaptic connections in neocortical networks. Nature 473, 97–91. 10.1038/nature09880 21478872 PMC3089591

[B39] Liley D. T. J. Wright J. J. (1994). Intracortical connectivity of pyramidal and stellate cells: estimates of synaptic densities and coupling symmetry. Network 5, 175–189. 10.1088/0954-898X_5_2_004

[B40] Lomo T. (2003). The discovery of long-term potentiation. Phil. Trans. R. Soc. Lond. B, 358, 617–620. 10.1098/rstb.2002.1226 12740104 PMC1693150

[B41] MacLean P. D. (1973). A triune concept of the brain and behavior, in The Hincks Memorial Lectures, eds Boag T. J. Campbell D. (Toronto, ON: University of Toronto Press), 6–66.

[B42] Mariño J. Schummers J. Lyon D. C. Schwabe L. Beck O. Wiesing P.. (2005). Invariant computations in local cortical networks with balanced excitation and inhibition. Nat. Neurosci. 8, 194–201. 10.1038/nn1391 15665876

[B43] Markram H. Tsodyks M. (1996). Redistribution of synaptic efficacy between neocortical pyramidal neurons. Nature 382, 807–810. 10.1038/382807a0 8752273

[B44] Martin K. A. C. Roth S. Rusch E. S. (2014). Superficial layer pyramidal cells communicate heterogeneously between multiple functional domains of cat primary visual cortex. Nat. Commun. 5, 5252. 10.1038/ncomms6252 25341917 PMC4354012

[B45] McGuire B. A. Gilbert C. D. Rivlin P. K. Wiesel T. N. (1991). Targets of horizontal connections in macaque primary visual cortex. J. Comp. Neurol. 305, 370–392. 10.1002/cne.903050303 1709953

[B46] Merker B. H. (2016). Cortical gamma oscillations: details of their genesis preclude a role in cognition. Front. Comput. Neurosci. 10:78. 10.3389/fncom.2016.00078 27512371 PMC4961686

[B47] Miyashita M. Tanaka S. (1992). A mathematical model for the self- organization of orientation columns in visual cortex. Neuroreport 3, 69–72. 10.1097/00001756-199201000-00018 1611037

[B48] Muir D. R. Da Costa N. M. A. Girardin C. C. Naaman S. Omer D. B. Ruesch E.. (2011). Embedding of cortical representations by the superficial patch system. Cereb. Cortex 21, 2244–2260. 10.1093/cercor/bhq290 21383233 PMC3169655

[B49] Muir D. R. Douglas R. J. (2011). From neural arbours to daisies. Cereb. Cortex 21, 1118–1133. 10.1093/cercor/bhq184 20884721 PMC3077431

[B50] Obermayer K. Blasdel G. G. (1993). Geometry of orientation and ocular dominance columns in monkey striate cortex. J. Neurosci. 13, 4114–4129. 8410181 10.1523/JNEUROSCI.13-10-04114.1993 PMC6576395

[B51] Obermayer K. Ritter H. Schulten K. (1990). A principle for the formation of the spatial structure of cortical feature maps. Proc. Natl. Acad. Sci. U.S.A. 87, 8345–8349. 10.1073/pnas.87.21.8345 2236045 PMC54952

[B52] Paik S.-B. Ringach D. L. (2011). Retinal origin of orientation maps in visual cortex. Nat. Neurosci. 14, 919–925. 10.1038/nn.2824 21623365 PMC3196663

[B53] Reimann M. W. King J. G. Muller E. B. Ramaswamy S. Markram H. (2015). An algorithm to predict the connectome of neural microcircuits. Front. Comput. Neurosci. 9:120. 10.3389/fncom.2015.00120 26500529 PMC4597796

[B54] Robinson P. A. Rennie C. J. Wright J. J. (1998). Synchronous oscillations in the cerebral cortex. Phys. Rev. E 57, 4578–4588. 10.1103/PhysRevE.57.4578

[B55] Rockland K. S. Lund J. S. (1983). Intrinsic laminar lattice connections in primate visual cortex. J. Comp. Neurol. 216, 303–318. 10.1002/cne.902160307 6306066

[B56] Sharma J. Angelucci A. Rao S. Sur M. (1995). Relationship of intrinsic connections of orientation maps in ferret primary visual cortex: iso-orientation domains and singularities, in Presented at Society for Neuroscience, (San Diego, CA).

[B57] Sherk H. Stryker M. P. (1976). Quantitative study of orientation selectivity in visually inexperienced kittens. J. Neurophysiol. 39, 63–70. 1249604 10.1152/jn.1976.39.1.63

[B58] Singer W. (1994). Putative functions of temporal correlations in neocortical processing, in Large-Scale Neuronal Theories of the Brain, eds Koch C. Davis J. J. (Cambridge, London: MIT Press), 201–237.

[B59] Singer W. (1999). Neuronal synchrony: a versatile code for the definition of relations? Neuron 24, 49–65. 10677026 10.1016/s0896-6273(00)80821-1

[B60] Swindale N. V. (1982). A model for the formation of orientation columns. Proc. R. Soc. Lond. B Biol. Sci. 215, 211–230. 10.1098/rspb.1982.0038 6127704

[B61] Swindale N. V. (1992). A model for the coordinated development of columnar systems in primate striate cortex. Biol. Cybern. 66, 217–230. 10.1007/BF00198475 1311609

[B62] Swindale N. V. (1996). The development of topography in the visual cortex: a review of models. Network 7, 161–247. 10.1088/0954-898X_7_2_002 16754382

[B63] Tanaka S. (1990). Theory of self- organization of cortical maps: mathematical framework. Neural Netw. 3, 625–640. 10.1016/0893-6080(90)90053-N

[B64] Tsukada M. Aihara T. Mizuro M. Kato H. Ito K. (1994). Temporal pattern sensitivity of long-term potentiation in hippocampal CA1 neurons. Biol. Cybern. 70, 495–503. 10.1007/BF00198802 7915144

[B65] Van Hooser S. D. Heimel J. A. Chung S. Nelson S. B. (2006). Lack of patchy horizontal connectivity in primary visual cortex of a mammal without orientation maps. J. Neurosci. 26, 7680–7692. 10.1523/JNEUROSCI.0108-06.2006 16855096 PMC6674269

[B66] Varela F. Lachaux J.-P. Rodriguez E. Martinerie J. (2001). The brainweb: phase synchronization and large-scale integration. Nat. Rev. Neurosci. 2, 229–239. 10.1038/35067550 11283746

[B67] von der Malsburg C. (1973). Self-organization of orientation sensitive cells in the striate cortex. Kybernetic 14, 85–100. 10.1007/BF00288907 4786750

[B68] Wiesel T. N. Hubel D. H. (1974). Ordered arrangement of orientation columns in monkeys lacking visual experience. J. Comp. Neurol. 158, 307–318. 10.1002/cne.901580306 4215829

[B69] Wright J. J. (2009). Generation and control of cortical gamma: findings from simulation at two scales. Neural Netw. 22, 373–384. 10.1016/j.neunet.2008.11.001 19095406

[B70] Wright J. J. (2010). Attractor dynamics and thermodynamic analogies in the cerebral cortex: synchronous oscillation, the background EEG, and the regulation of attention. Bull. Math. Biol. 73, 436–457. 10.1007/s11538-010-9562-z 20821066

[B71] Wright J. J. Bourke P. D. (2013). On the dynamics of cortical development: synchrony and synaptic self-organization. Front. Comput. Neurosci. 7:4. 10.3389/fncom.2013.00004 23596410 PMC3573321

[B72] Wright J. J. Bourke P. D. Chapman C. L. (2000). Synchronous oscillation in the cerebral cortex and object coherence: simulation of basic electrophysiological findings. Biol. Cybern. 83, 341–353. 10.1007/s004220000155 11039699

[B73] Wright J. J. Bourke P. D. Favorov O. V. (2014). Möbius-strip-like columnar functional connections are revealed in somato-sensory receptive field centroids. Front. Neuroanat. 8:119. 10.3389/fnana.2014.00119 25400552 PMC4215792

[B74] Wright J. J. Liley D. T. J. (1996). Dynamics of the brain at global and microscopic scales: neural networks and the EEG. Behav. Brain Sci. 19, 285–295.

[B75] Wu S. Wong K. Y. M. Tsodyks M. (2013). Neural information processing with dynamical synapses. Front. Comput. Neurosci. 7:188. 10.3389/fncom.2013.00188 24421767 PMC3872650

[B76] Yousef T. Tóth É. Rausch M. Eysel U. T. Kisvárday Z. F. (2001). Topography of orientation center connections in the primary visual cortex of the cat. Neuroreport 12, 1693–1699. 10.1097/00001756-200106130-00035 11409741

[B77] Zucker R. Regehr W. (2002). Short-term synaptic plasticity. Annu. Rev. Physiol. 64, 355–405. 10.1146/annurev.physiol.64.092501.114547 11826273

